# Informal caregivers of children with cerebral palsy ways of coping with the uncertainty of illness^*^

**DOI:** 10.1590/1980-220X-REEUSP-2023-0115en

**Published:** 2023-12-15

**Authors:** Isabella Joyce Silva de Almeida Carvalho, Inácia Sátiro Xavier de França, Francisco Stélio de Sousa, Francisca Márcia Pereira Linhares, Ana Luisa Brandão de Carvalho Lira, Alexsandro Silva Coura

**Affiliations:** 1 Universidade de Pernambuco, Departamento de Enfermagem, Petrolina, PE, Brazil.; 2 Universidade Estadual da Paraíba, Departamento de Enfermagem, Campina Grande, PA, Brazil.; 3 Universidade Federal de Pernambuco, Departamento de Enfermagem, Recife, PE, Brazil.; 4 Universidade Federal do Ceará, Departamento de Enfermagem, Fortaleza, CE, Brazil.

**Keywords:** Caregivers, Cerebral Palsy, Nursing Theory, Health Promotion, Cuidadores, Parálisis Cerebral, Teoría de Enfermería, Promoción de la Salud, Cuidadores, Paralisia Cerebral, Teoria de Enfermagem, Promoção da Saúde

## Abstract

**Objective:**

To unveil the process of collective construction of interventions for coping by informal caregivers of children with cerebral palsy using the Theory of Uncertainty in Illness.

**Method:**

Qualitative action-research in a hybrid format with informal caregivers of children with cerebral palsy registered with the Raros group in Petrolina, Pernambuco. The research followed the planned intervention cycle, going through four phases. The analysis was carried out using the IRAMUTEQ software and content analysis.

**Results:**

Interventions were designed collectively, both virtually and in person, which resulted in improvements for informal caregivers in coping with the conditions associated with the disability, promotion of self-care, empowerment and the construction of a sense of belonging to the group. There were 12 participants, all of whom were mothers.

**Conclusion:**

There was a facilitation of the process of coping with uncertainty in the disease on the part of the informal caregiver of children with cerebral palsy and it was evidenced that for this a prismatic perspective is necessary, which understands that the uncertainties are not only related to the conditions associated with cerebral palsy, but involve subjective aspects of the caregivers.

## RESUMO

**Objetivo:**

Desvelar o processo de construção coletiva de intervenções para o enfrentamento pelo cuidador informal de crianças com paralisia cerebral, à luz da Teoria da Incerteza na Doença.

**Método:**

Pesquisa-ação, de abordagem qualitativa, no formato híbrido, com os cuidadores informais de crianças com paralisia cerebral cadastrados no grupo Raros em Petrolina, Pernambuco. A pesquisa seguiu o ciclo de intervenção planejada, percorrendo quatro fases. A análise contou com o auxílio do *software* IRAMUTEQ e da análise de conteúdo.

**Resultados:**

Coletivamente foram concebidas intervenções, na modalidade virtual e presencial, que resultaram para os cuidadores informais em melhorias do enfrentamento às condições associadas à deficiência, promoção do autocuidado, empoderamento e construção de significado de pertença ao grupo. Houve 12 participantes, sendo todas elas mães.

**Conclusão:**

Houve uma facilitação do processo de enfrentamento de incerteza na doença por parte do cuidador informal de crianças com paralisia cerebral e evidenciou-se que para isso é necessária uma ótica prismática, que compreenda que as incertezas não são relacionadas apenas às condições associadas à paralisia cerebral, mas envolvem aspectos subjetivos dos cuidadores.

## RESUMEN

**Objetivo:**

Desvelar el proceso de construcción colectiva de intervenciones de afrontamiento por cuidadores informales de niños con parálisis cerebral a la luz de la Teoría de la Incertidumbre en la Enfermedad.

**Método:**

Investigación-acción cualitativa en formato híbrido con cuidadores informales de niños con parálisis cerebral inscritos en el grupo Raros, en Petrolina, Pernambuco. La investigación siguió el ciclo de intervención previsto, pasando por cuatro fases. El análisis se realizó mediante el software IRAMUTEQ y análisis de contenido.

**Resultados:**

Se diseñaron intervenciones colectivas, virtuales y presenciales, que resultaron en mejoras para los cuidadores informales en el afrontamiento de las condiciones asociadas a la discapacidad, la promoción del autocuidado, el empoderamiento y la construcción de un sentido de pertenencia al grupo. Participaron 12 personas, todas ellas madres.

**Conclusión:**

Hubo una facilitación del proceso de afrontamiento de la incertidumbre en la enfermedad por parte de los cuidadores informales de niños con parálisis cerebral y se evidenció que esto requiere una perspectiva prismática, que entienda que las incertidumbres no se relacionan solamente con las condiciones asociadas a la parálisis cerebral, sino que involucran aspectos subjetivos de los cuidadores.

## INTRODUCTION

Cerebral Palsy (CP) affects 1 in 500 newborns, presenting a prevalence of 17 million people worldwide. It is defined as a multifactorial, non-progressive disease whose repercussions are linked to the involvement of the Central Nervous System. It is associated with prenatal, perinatal and postnatal factors, such as prematurity, low birth weight and neonatal asphyxia. It has associated comorbidities such as visual, hearing and intellectual impairment, behavioral disorders and, most commonly, epilepsy^(1,2,3)^.

In this sense, caring for a child with CP unveils a routine full of challenges, such as a change in family dynamics, with possible strain on parental relationships; distressing feelings such as stress and anguish, which directly affect family well-being; restrictions on social life; and economic vulnerability^(3,4)^.

Thus, it is usually the mother who takes on the role of ­caregiver, and is thus classified as an informal caregiver, as she provides non-professional care without receiving any remuneration for it^(5,6)^.

In this way, informal caregivers, along with the family, are placed in a delicate situation, ranging from the deconstruction of the “perfect” child, the difficulty of understanding the diagnosis, ineffective communication with health professionals, the feeling of denial of reality and, finally, the process of adapting to the child’s special needs^(7)^.

The adaptation process is guided by coping. In turn, coping becomes positive when there is less uncertainty. From this ­perspective, Merle Mishel, author of the Theory of Uncertainty in Illness, proposed three main points that make it possible to recognize the genesis of uncertainty and its respective modes of coping: the antecedents of uncertainty; the evaluation of uncertainty; and coping strategies. This is possible through the implementation of dialogical, humanized and horizontal health education interventions, supported by meaningful learning, which takes into account the learner’s previous knowledge and worldview^(8–10)^.

Concurring with this concept, one study identified the need to develop interventions that foster spaces for welcoming and sharing, with the aim of promoting health and strengthening the caregiver’s coping strategies^(11)^.

In view of the above, it can be inferred that the informal caregiver is immersed in an unknown universe full of challenges in the context of cerebral palsy (disease). Therefore, it is necessary to recognize this reality in order to intervene, thus fostering an adequate adaptation of the informal caregiver. For this reason, the study aims to unveil the process of collective construction of interventions for informal caregivers of children with CP to cope with the uncertainty of the disease.

## METHOD

### Type of Study

This is an action-research study with a qualitative approach^(12)^.

### Study Setting

The study took place in a hybrid way, permeating between the virtual environment, through the tools of the company Google®, and at the headquarters of the Sociedade Integrada de Pessoas Com Síndromes e Doenças Raras, Famílias e Amigos do Vale do São Francisco (Integrated Society of People with Rare Syndromes and Diseases, Families and Friends of the São Francisco Valley), also known as Grupo Raros, in Petrolina, Pernambuco (PE), Brazil, on the occasion of the COVID-19 pandemic.

### Participants

All 36 informal caregivers of children with CP registered with the Raros Group were included in the study. However, the final number of participants was 12, because three (03) had technical training in the health area (exclusion criterion), 10 caregivers did not participate in all stages of the research (loss criterion) and the rest (11 caregivers) chose not to participate in the research.

### Study Protocol

In order to carry out the research, an extension project entitled “Caring for those who care” was created through a continuous flow public notice issued by the Pro-Dean for Extension and Culture (PROEC) at the University of Pernambuco (UPE). All the interventions involved the participants and the research team, made up of the researcher in charge and the extension project team.

The action-research was structured by the “Entry into the Field” moment, where the researcher began the previous interaction by means of a telephone call and participation in the Raros WhatsApp group, and then the research was guided by the planned intervention cycle^(13)^, in the light of Merle Mishel’s theory of uncertainty in illness, as detailed below (Chart 1).

**Chart 1:** Summary of the phases of the Planned Intervention Cycle. Source: Authors – Petrolina, PE, Brazil, 2022.

Phase/Period	Activities carried out
1st Phase 04/21 –07/21	– Implementation of online focus groups, through the extension project “Caring for those who care”, with the aim of uncovering the “Cognitive Schemes” that generate uncertainty in the participants. They lasted an average of one and a half hours; – Collection of information aimed at tracing the socio-economic profile of the participants.
2nd Phase 08/21	– Presentation of the results of the analysis of the speeches of the 1st phase, which were extracted by transcribing the audios of the recordings of the online focus groups, via google meet; – Group discussion using the brainstorming technique, which includes the creative phase and the critical phase; – Presentation of suggestions for resolving the demands identified in the 1st phase (creative phase); – Definition of the best suggestions for resolution (critical phase); – Collaborative construction of the intervention plan.
3rd Phase 09/21–01/22	– Implementation of the intervention plan through hybrid actions. – Interventions that took place virtually: Creation of the WhatsApp group for the “Caring for those who Care” project; 1st Cycle of Debates - Caring for those who Care; Cycle of Debates - Acceptance. – Interventions that took place in person: 1st Workshop - Dealing with conditions associated with disability; Wellness Tea; and Children’s Day Campaign/Children’s Day Party.
4th Phase 04/22	– Implementation of the focus group three months after the 3rd phase, with the triggering question: “What was it like for you to take part in this study and what changes has it brought to your life?”

### Study Analysis

The study used the Interface de R pour les Analyses Multi­dimensionnelles de Textes et de Questionnaires (IRAMUTEQ) software version 0.7 alpha 2 to analyze the focus groups in the 1st phase of the study, with simple classification of text segments, using Descending Hierarchical Classification^(14)^, which categorizes the most significant words into classes, which are defined by applying chi-square tests. The analysis of the fourth phase was carried out using Thematic Content Analysis^(15)^, comprising the stages of pre-analysis; exploration of the material; and treatment of the results, inference and interpretation. The classes that emerged from both analyses were named in the light of the Theory of Uncertainty in Illness and the relevant scientific literature^(7)^.

### Ethical Aspects

The research is in line with Resolution 466/12, used the Free and Informed Consent Form and was cleared by the Research Ethics Committee of the Osvaldo Cruz University Hospital Complex and Cardiology Emergency Room of Pernambuco, opinion 5.782.963, approved in 2021. To preserve the identities of the participants, they were identified by flower codenames.

## RESULTS

The study included 12 participants, all of whom had a maternal bond with the child; nine caregivers (09) received financial assistance from the social security system; eight (08) stated that the child attends school; five (05) reported that the child has seizures routinely; two (02) shared that the child has episodes of choking; four (04) said that the child suffers from episodes of falls. Each child uses an average of three (03) medications every day and four (04) caregivers reported having difficulty managing their child’s medication.

To make it easier to understand the results, they were structured in the light of the planned intervention cycle^(13)^.

### 1° Phase: Identification of Initial Situations

After analyzing the statements, with a retention level of 83.16%, six classes emerged, as shown below (Chart 2).

**Chart 2:** First phase analytic dendrogram. Source: Authors – Petrolina, PE, Brazil, 2022.

Class 1 (15,2%)	Class 4 (17,1%)	Class 5 (21,5%)	Class 3 (15,2%)	Class 2 (12%)	Class 6 (19%)
Car	Taking	Important	Home	Good	Receive
Health	Anxiety	Mother	Fear	Gratifying	Need
Choking	Medication	Group	Inside	Participating	Life
Child	Time	Watch	Learning	Night	Never
Neighbor	Seizure	After	Want	Always	Entitlement
Help	Convulsive	Disability	Thing	Talk	Benefit
Calm	Doubt	Fighting	People	Time	Company
Save	Effect	Call	Today	Here	Son
Attention	Control	Alone	Prepare	Project	Salary
Episode	Happening	Importance	Plenty	Story	Reality
Constantly	Response	Very Important	World	Team	Public
Doctor	Change	Know	Less	Thank you	Living
Ask	Generate	Interesting	Share	Listen	Work
Year	Still	Interesting	Good	Also	Question
Case	Stay	Child	Even	Girl	Caring
	Anguish	Find	Leave	Give	Thinking
				Space	

In this phase, it was possible to identify the participants’ cognitive schemas that generate the “Antecedents of uncertainty” and thus carry out the “Evaluation of uncertainty” according to the imaginary brought up by the participants’ speeches. This shows that emergency episodes are a source of great concern; that feelings related to the routine of care also play a part in the construction of this imaginary; that when they think about the context of care, they always associate a sense of belonging with the “Raros group” and the “Cuidar de quem Cuida (Caring for those who care) group”; and finally, they associate their dissatisfaction with access to rights.

As recommended by the dendrogram for class analysis and discussion, the partitions were followed from left to right. Classes 4, 5 and 3 had to be grouped together due to the closeness of the nuclei of ideas.

With regard to class 1, called “Emergency Episodes”, the statements brought back memories of emergency situations experienced. Many of these are related to conditions associated with disability, mainly episodes of suffocation and seizures. The most significant words in this class were “choking” and “seizure”.

*In 2017, she had a choking episode, I think due to reflux, where she lost consciousness and I couldn’t see her breathing, she didn’t react, so it was a very big choking (...) it was one of the worst days of my life (Rosa).*

*He had convulsive seizures every half an hour, it’s really distressing because I keep wondering if he’s in pain... (Cacto).*

With regard to Classes 4, 5 and 3, named “Feelings in relation to the universe of care”, the statements go back to what the caregivers feel within the care routine. They mainly identified anguish, anxiety and fear in relation to the situations associated with the disability, in relation to the future, in relation to the opinion of others and the overload of activities. The most significant words in this class were “anguish” and “anxiety”.

*A mother of a disabled child has to have a strong emotional state, because it’s a struggle (Violeta).*

*I worry a lot about what it will be like when I die, it makes me anxious and with anguish (Girassol).*

*We live for our children, everything for them has to be perfect, they have to be well fed, in order, but not us, we go out all messy, we go out barefoot, we go out hungry (Lirio).*

Regarding Class 2, named “Feelings in relation to participating in groups”, the statements reveal the extent to which this participation allows carers to recognize universes similar to their own, where a powerful support network can be woven. The most significant words in this class were “welcome”, “group” and “talk”.

*After I got to know the Raros group I joined, it changed me, I see that I’m not alone, that there are mothers who also have problems and even more than mine, and then we feel more welcomed (Papoula).*

*You created this space so that we could talk, chat, exchange experiences, because in a way the mothers’ stories motivate us (Lisianto).*

Regarding Class 6, named “Rights and social struggles”, the speeches show that they are subjected to social judgments that impose flagrant stigmas, as well as feeling that they are not covered by the legal frameworks imposed by the public authorities. The most significant words in this class were “benefit” and “society”

*We end up getting used to being called ‘mommy’, and by the time we realize it, we don’t even know who it is (Lisianto).*

*I see this a lot, that society still sees us as the mother of the family (Rosa).*

*Something that has always bothered me, since the beginning of the group, this issue that in order for our children to have the right to receive a benefit, we need to give up our benefits, our rights (Violeta).*

### 2° Phase: Planning Actions

After recognizing the “Antecedents of uncertainty” and the “Evaluation of uncertainty”, the “Coping strategies” construct could be worked out in this phase.

Based on the analysis of the speeches generated in the first phase, the group collectively composed interventions anchored in the constructs “Providers of structure”, “Cognitive capacity” and “Inference” of the Uncertainty in Illness theory. The “Intervention Plan” was thus drawn up, as detailed below:

1st What are the objectives of the action? To create a favorable space for promoting biopsychosocial health for caregivers of people with disabilities in order to help them cope with uncertainty. 2nd Who will carry out the plan? The Caring for Caregivers extension project in partnership with the Raros group.

3º How will we achieve the objectives? Through the interventions programmed in the 2nd phase and implemented in the 3rd phase. 4) How will we continue with the plan after this research has finished? By continuing the partnership between the Raros Group and the “Caring for those who care” extension project.

With regard to the “I Cycle of Debates - Caring for those who Care” and the “Cycle of Debates - Acceptance”, they were designed to help respond to the demands identified in Class 6 of the dendrogram, “Rights and social struggles”. Regarding the Children’s Day Campaign/Children’s Day Party, it was designed with the aim of reactivating the actions of the Raros Group, which due to the pandemic context, had its actions stopped, legitimizing the conceptions identified in Class 2 “Feelings in relation to group participation”.

With regard to the “1st Workshop - Dealing with conditions associated with disability”, it was conceived with the intention of contemplating the propositions envisioned in Class 1, “Episodes of Urgency”. Finally, the “Well-being Tea” aimed to encourage the beginning of the weaving of spaces that make up the fabric that involves the promotion of biopsychosocial health, in response to what was identified in classes 4, 5 and 3, “Feelings in relation to the universe of care”.

### 3ª Phase: Carrying Out the Planned Actions

The WhatsApp group was the first action implemented, with the aim of strengthening and facilitating communication between the research team and the participants. With regard to the events, the “1st cycle of debates - Caring for those who care”, took place on October 22 and 23, 2021, in online mode, where aspects of the rights and duties of people with disabilities were worked on; reflections on what it is like to be an informal caregiver for a person with a disability and its social and emotional repercussions. The event was broadcast on YouTube using the free version of the Streamyard platform.

Following on from the planned actions was the “Children’s Day Campaign/Children’s Day Party”. This event marked the Raros Group’s face-to-face activities and aimed to raise funds for the purchase and refurbishment of wheelchairs, which would be delivered at the Children’s Day party, which took place on October 30 and was broadcast on YouTube and Instagram.

As for the “Cycle of Debates - Acceptance”, this was an online event where people discussed coping strategies and shared situations aimed at strengthening the audience. This action was broadcast on YouTube, using the Streamyard tool, on November 5, 2021, with the effective participation of caregivers who interacted with the facilitators via chat.

As for the “1st Workshop - Dealing with conditions associated with disability”, it took place in person at the Raros Group headquarters on November 28, 2021. This action marked the beginning of the partnership between the “Caring for those who care” extension project and UNIVASF’s “Medicines Information Center” (CIM).

The workshop took a theoretical and practical approach to the demands associated with disability, namely suffocation episodes, seizures and medication management. The methodology used followed the postulates of meaningful learning.

While there was a brief dialogical presentation on the theoretical aspects of the associated conditions, it was possible to anchor this dialog in the shared previous experiences. This was followed by a simulation of maneuvers in cases of suffocation and seizures, in which the caregivers took part in care simulation activities. In addition, there was a “truth or lie” dynamic, related to the topics covered and, finally, the CIM/UNIVASF team started a conversation about medication management.

The “Wellness Tea” took place in person on January 9, 2022, at the headquarters of the Raros group in Petrolina-PE. The action aimed to foster a space for promoting biopsychosocial health for caregivers.

The intervention began with a coffee break, followed by a round table discussion on the theme “Despite... You have to know how to live well”. The environment was carefully prepared to welcome the participants, and the action began with the song “one day after the other” by singer Tiago Iorc. The next step was a provocation about how to recognize escape routes in the care routine that promote the caregiver’s health and well-being.

After this, the participants were given sheets of paper and wrote down self-care “promises” they were going to make to themselves. They then went to stands offering services such as auriculo-puncture, anthropometric assessment and blood pressure measurement.

### 4ª Phase: Evaluation of the Results Obtained

This phase was analyzed according to thematic content analysis, in the light of the “Adaptation” construct of the theory of uncertainty in illness, where the following categories emerged: 1) Improvement in coping with conditions associated with disability; 2) Self-care and empowerment; and 3) Meanings in belonging to the group, which will be revealed below.

Regarding the category “Improvement in coping with conditions associated with disability”, the statements reveal that the caregivers feel safer in certain situations related to caring for their child, especially in relation to episodes of choking, medication management and seizures. The most significant words in this class were “prepared” and “notion”.

*With regard to seizures and medication, it was very important for me because I had a lot of doubts and I was able to clear them up... (Tulipa).*

*... I feel prepared at that moment to resolve the situation, to find a solution, without stress, without getting nervous (Girassol).*

With regard to category 2 “Self-care and empowerment”, the speeches go back to a recognition in which they noted that in order to take good care of their child, they need to take good care of themselves, thus weaving speeches that denote attitudes of self-care and empowerment. The most significant words in this class were “care” and “importance”.

*With this last meeting we had in person, where we had to write down on paper what we would like to do... I’m going to try to develop it, considering that I can’t leave the house, I’ve developed it at home, I’m doing physical exercise, stretching, which I didn’t used to do (Lisianto).*

*... There are people who remind us of our importance, who remind us that we can, that we need and who we are (...) I have to look after myself to look after someone else, because if I don’t it’s going to get complicated down the line (Sunflower).*

*... Today I’m trying to get back to myself a bit. I try to look after myself, because I’m also going to be looking after him (...) (Cacto).*

In the third category “Meanings of belonging to the group”, the speeches show the caregivers’ perspective on the actions of the “caring for those who care” group, celebrating the fact that there are actions aimed at the caregiver, who is often forgotten by society. The most significant words in this class were “grateful” and “happy”.

*We’re happy that people who don’t have atypical children are concerned about us, because it’s rare (Lisianto).*

*... I’m very grateful to be part of this team (...) it’s very important for our development both as mothers and as people, because we learn so much from exchanging experiences and from these meetings... (Tulipa).*

## DISCUSSION

This study, in the light of a mid-range nursing theory, also legitimizes the profession as a science. It is nursing care, whether in care, research, teaching and/or management, based on a solid theoretical framework, which enables nurses to practice in an instrumentalized, systematized, critical, reflective, ethical and holistic way^(16)^.

In this sense, other research carried out by nurses links the theory of uncertainty in illness with other objects of study, such as the study carried out in Lisbon, which focused on understanding how uncertainty in illness can mediate the communication process between nurses and families^(17)^.

Therefore, the discussion was structured in the light of the theory of uncertainty in illness, using the constructs “cognitive schema”; “structure providers”; and “adaptation” and the relevant literature on the subject. The “cognitive schema” construct is defined as mental organization in the context of the illness, taking into account the subjective aspects of the individual^(9)^. Thus, the cognitive schema could be glimpsed in the implementation of the first phase of this research.

With regard to the socio-economic background of the participants, all were female. This is in line with a study carried out in Pará, where there is a predominance of females in the role of caregiver for children with CP^(18)^.

“Class 1: Emergency Episodes”, on the other hand, brings situations that immerse the caregiver in a universe full of uncertainties. It is also known that the level of knowledge about the disease interacts as a predictor of caregiver burden, directly influencing their quality of life^(8,19)^. According to the Uncertainty in Illness theory, this is due to the inability to identify the pattern of symptoms and familiarity with the event^(9)^.

With regard to “Classes 4, 5 and 3: Feelings in relation to the universe of care”, the statements corroborate another study which indicates feelings such as an overload of activities, since they dedicate themselves entirely to care, thus bringing economic, physical and/or emotional problems which directly alter their quality of life; difficulties in accessing the job market; and physical and emotional exhaustion^(20)^.

Regarding “Class 2: Feelings in relation to participation in groups and associations”, the speeches refer to the importance of support networks, which consists of the peculiar relationships that are established, based on trust, sharing and acceptance. This reality is relevant, especially when you consider that there is greater stress on the part of the caregiver, while there is a low level of social support experienced during the care process^(21)^.

With regard to “Class 6: Rights and social struggles”, the speeches are in line with studies which identify that public policies are unable to meet the real demands of this world of care, especially when it comes to the rights of the caregiver. In this sense, caregivers need to build a critical political notion, because, supported by the biomedical model, they associate access to rights only with access to health services, because they are unaware of their legal protections^(22,23)^.

With regard to the construct “structure providers”, which was covered by the implementation of the 2nd and 3rd phases of the study, it is referred to as resources that help the subject to interpret the stimuli (of the disease), guiding their appreciation and, consequently, interacting directly in their adaptation^(9)^.

From this perspective, action research stands out, as it provides an interlocution between popular and academic knowledge, with the aim of solving problems related to the practical context, bringing protagonism and autonomy to the individuals who belong to this scenario^(12)^. From this perspective, Paulo Freire stresses the need to raise awareness for social transformation, which transgresses models of domination and weaves autonomy through horizontal, emancipatory and dialogical constructions of knowledge^(10)^.

With regard to the third theoretical construct, “Adaptation”, it is defined as the result of effective coping, where the individual, faced with the facts inherent in the disease, achieves a biopsychosocial balance^(9)^. This construct is contemplated through the experience of the 4th phase of this study.

Studies corroborate the findings of this research, where it is revealed that educational interventions aimed at caregivers can empower them in the face of their repressed learning needs, improving their care skills, as well as making them multipliers of knowledge with their peers^(24,25)^.

With regard to the category “Self-care and empowerment”, the awakening of a naïve awareness to a critical awareness is identified. In line with the interventions that took place in this study, a study carried out in Turkey subjected caregivers of people with cancer to an empowerment program via cell phone, in which caregivers submitted to the intervention group had a lower distress score and higher quality of life scores^(26)^.

Next, the category “Meanings of belonging to the group” emerged. It is well known that caregivers feel lonely. This reality has been made worse by the COVID-19 pandemic, as a study carried out in Canada revealed that there was an 85.9% increase in feelings of loneliness and a 78.8% increase in anxiety among the public in question^(27,28)^.

As such, loneliness directly affects the mental, emotional and physical well-being of caregivers. Therefore, social support is linked to improved quality of life, especially interaction with peers, due to the possibility of sharing similar situations and feelings^(29)^.

For this reason, this study reveals the need for a humanized, dialogical, horizontal and holistic approach to the public in question, because it is by caring for those who care for them that we can conceive an effective way of dealing with the uncertainties in the world of caring for children with CP.

The limitations of this study are the impossibility of implementing all the phases of the research in person, due to the COVID-19 pandemic. In addition, the hybrid format required access to certain consumer goods such as the internet, smartphones or laptops, which not all caregivers had.

## CONCLUSION

By identifying cognitive schemas in order to offer structure providers, with the aim of promoting caregiver adaptation, through the stages of this study, it was revealed that coping with uncertainties in the illness of a child with CP requires a prismatic perspective, which understands that uncertainties are not only related to conditions associated with CP, but also involve subjective aspects of caregivers.

Therefore, through the construction of the intervention plan, there was a facilitation of the process of coping with uncertainties of the informal caregiver of children with CP, which promotes health and well-being for caregivers, while fostering actions of empowerment and self-care, construction of knowledge about the conditions associated with CP and participation in groups and associations by them, which configures an adequate adaptation, as recommended by the theoretical framework used in this research.

Thus, this study contributes to the construction of a broader view of what is uncertainty in the illness in the universe of children with CP, from the perspective of the informal caregiver, gives visibility to this public and can help subsidize the construction of public policies that include the demands of the caregiver.

Finally, we suggest developing studies using participatory methodology in other groups or associations that have informal caregivers, in order to learn about different realities and thus intervene, with the aim of promoting the health of these people.

## Data Availability

Supplementary material available via DOI and/or URL: 10.17605/OSF.IO/Y5CNF - https://osf.io/y5cnf/.
